# Commentary: Environmental Influences on Elite Sport Athletes Well Being: From Gold, Silver, and Bronze to Blue, Green and Gold

**DOI:** 10.3389/fpsyg.2017.00078

**Published:** 2017-01-31

**Authors:** Mike Rogerson

**Affiliations:** Centre for Sports and Exercise Science, School of Biological Sciences, University of EssexColchester, UK

**Keywords:** well being, olympic games, mental health, elite athletes, air pollution, physical activity, greenspace, green exercise

## Introduction

The article “Environmental Influences on Elite Sport Athletes Well Being: From Gold, Silver, and Bronze to Blue, Green and Gold” (EIEPAWB) pioneeringly addresses a range of environmental health aspects in relation to elite sports performance and athletes' general health and well-being. It highlights the importance of environment across both sides of an often-prominent split in the field of sports and exercise science: the respective domains of *sports performance*, and *exercise and health*. Specifically, the Olympics represent a relevant, yet little-explored arena, to be examined in terms of environmental affects. Addressing this may serve two purposes. Firstly, it may be of use to elite sporting performance. Secondly, Olympic sport offers a publicly known and valued vehicle for raising awareness of health impacts of greener environments. This commentary draws attention to some of the important points made by EIEPAWB, and offers further thoughts both on the application of environmental influences for elite sports performance, and on these three domains linked by the Olympics: environment, sports performance and health.

### Environmental affects for sports training and performance

Environmental pollution and the aesthetically perceived environment can both impact on sports performance. The Olympic games represents the pinnacle of sports performance, with differences between gold medal performance and no medal performance being as slight as fractions of a second. Just as equipment, facilities and infrastructure are designed for optimal performance, athletes, host cities, sponsors and spectators alike should have interest in optimizing environmental variables toward enhancing training and performance.

Acute physiological responses to atmospheric environmental pollutants detrimentally impact sports performance. Given the orientation of athletes' training toward reaching physiological peak with precise timing, it is somewhat surprising that such detrimental physiological impacts are often readily accepted once residing within host cities that are high in specific and total suspended particles. Doses of exposure to nature and green exercise bring performance-relevant physiological, immunological, and psychological benefits. Therefore, beyond the use of other methods such as altitude training and oxygen chambers for recovery and training gains, residing and training in greenspaces away from the host city immediately prior to and during competition could avoid detrimental impact of host city pollution.

EIEPAWB outlines theory and evidence that both exercising in, and simple exposure to nature environments can both function to improve affective state, and reduce stress and aid recovery from stressful events. Although environmental-arousal-performance dynamics are likely to vary between sports (Hanton et al., 2000), an interesting prospect here, is how these environmental influences on performance-related stress might function in conjunction with arousal theories of sports performance; deliberate strategies to reach optimal arousal levels for either training or performance may benefit from environmental considerations.

EIEPAWB also outlines evidence that greenspaces and perceived restorative aspects of environmental settings can function to reduce perceived exertion and directly improve athletic performance. Additionally to other relevant perceptual, psychological, physiological, and psychophysiological influences of exercise environments (Thompson Coon et al., 2011; Aspinall et al., 2015; Rogerson et al., 2016), a key component of training in greenspaces is that movement through these environments typically creates optic flow of large extent, whereas ergometer-based indoor exercise usually does not. Alongside other factors such as prior experience, optic flow functions as an important cue for an individual's cognitive “performance template” (Tucker and Noakes, 2009), which is used to assess fatigue and exertion in relation to performance expectations. Environmental settings offer a tool for manipulating or deceiving athletes' training and performance experiences. A simple example of this might be in an instance whereby an athlete reports feeling generally fatigued from their training program, but their perception is not substantiated by collected physiological data; via prescribed exercise intensity using a Rated Perceived Exertion Scale, a coach may use environmental settings to “dupe” an athlete into working physiologically harder than they perceive. When taking a holistic approach to athletes and their training, nature environments can also contribute positively to athletes' psychological state and well-being. Therefore, combining environmental manipulations with physiology and performance data may afford new possibilities for coaches to better design training sessions and programs for optimal physiological and psychological impact.

### Public interest in olympic-related valuing of greener environments

Olympic sport offers a publicly known and valued vehicle by which to raise awareness of the health impacts of greener environments. Commercial sports product manufacturers target the Olympic games, because elite sport sponsorship and endorsement programmes link directly to product sales (DeGaris and West, 2012). When the general public observe athletes using and valuing specific products for training, performance or recovery gains, they accept their efficacy—often then purchasing products and altering behaviors.

There remains a paucity of evidence that hosting an Olympic games stimulates increased participation in physical or sporting activities, and little evidence to suggest other health benefits (Mahtani et al., 2013). Here, that greener environments can benefit athletes' health, well-being and performance, alludes that endorsement of this from the elite sports community could offer a vehicle for increasing greenspace visits and green exercise participation—thereby enhancing public health. To speculate, sports celebrities may be used as faces of public health campaigns to raise awareness of the health and well-being benefits of the aesthetic beauty and meaningfulness of activity afforded by greenspaces. Additionally, individuals who are interested in sports performance are likely to follow practices that they feel may enhance their own performance. Alongside the positive health impacts of visiting greenspaces or utilizing them for exercise, there are some barriers, such as access to greenspace, and health and safety risks ranging from tick and snake bites, to muggings in parks. These considerations place emphasis on local authorities to ensure that issues of this kind do not hinder any such Olympic-inspired public health drives.

## Conclusions

There are a number of potential elite sporting applications that emanate from research findings discussed in EIEPAWB, relating to existing research in the domains of environment, health, well-being, exercise and performance. There is also a possible pathway linking the Olympics with population health—that is, the valuing of greener environments.

## Author contributions

The author confirms being the sole contributor of this work and approved it for publication.

### Conflict of interest statement

The author declares that the research was conducted in the absence of any commercial or financial relationships that could be construed as a potential conflict of interest.
